# Low temperature superplasticity and thermal stability of a nanostructured low-carbon microalloyed steel

**DOI:** 10.1038/srep18656

**Published:** 2015-12-21

**Authors:** J. Hu, L.-X. Du, G.-S. Sun, H. Xie, R.D.K. Misra

**Affiliations:** 1 The State Key Laboratory of Rolling and Automation, Northeastern University, Shenyang 110819, China; 2 Laboratory for Excellence in Advanced Steel Research, Metallurgical and Materials Engineering Program, Department of Metallurgical, Materials and Biomedical Engineering University of Texas at El Paso, TX 79968-0521, USA

## Abstract

We describe here for the first time the low temperature superplasticity of nanostructured low carbon steel (microalloyed with V, N, Mn, Al, Si, and Ni). Low carbon nanograined/ultrafine-grained (NG/UFG) bulk steel was processed using a combination of cold-rolling and annealing of martensite. The complex microstructure of NG/UFG ferrite and 50–80 nm cementite exhibited high thermal stability at 500 °C with low temperature elongation exceeding 100% (at less than 0.5 of the absolute melting point) as compared to the conventional fine-grained (FG) counterpart. The low temperature superplasticity is adequate to form complex components. Moreover, the low strength during hot processing is favorable for decreasing the spring back and minimize die loss.

Superplasticity is characterized as the ability of the material to sustain large plastic strain prior to failure^1^,^2^,^3^, which is well documented for titanium alloys^4^,^5^,^6^. Among the various strengthening mechanisms in metals, grain refinement is considered as the appropriate and convenient method to simultaneously improve both strength and toughness^7^,^8^,^9^,^10^,^11^. Nanostructured stainless steels processed via a combination of severe cold deformation (>65%) followed by phase reversion annealing exhibited superior strength-ductility combination including high strength/weight ratio, wear resistance, and also favorable cellular activity. In this approach, severe deformation of metastable austenite at room temperature leads to strain-induced transformation of austenite to martensite. On annealing, this severely deformed strain-induced martensite reverts back to austenite either via a martensitic shear or diffusional reversion mechanism^12^,^13^,^14^. Thus, there exists a strong potential for using nanostructured stainless steels in lieu of the conventional coarse-grained counterpart. However, in majority of the nanostructured alloy systems, ductility is limited because of lack of strain hardening ability^3^,^4^,^5^,^6^,^7^,^8^,^9^,^10^,^11^,^12^,^13^,^14^,^15^,^16^. Thus, improvement in plasticity continues to be the subject of interest. Attention has also been given to high temperature superplasticity to form complex shaped components and curved parts through grain boundary engineering^2^,^17^. In ultrafine-grained (UFG) materials, grain boundary sliding is considered as the most important mechanism of superplasticity. Fine grain size is preferred because the number of grain boundaries involved in sliding is high and the distance for accommodation by diffusion and/or slip is small^18^. We explore here superplasticity in microalloyed low carbon nanograined/ultrafine-grained (NG/UFG) bulk steel at elevated temperature, but significantly below 0.5 melting point (Tm).

There is no doubt that the application field of superplastic forming will become even more widespread if the deformation temperatures can be lowered because of lower energy consumption and significant reduction in surface oxidation. The superplasticity of nanocrystalline nickel was observed at 470 °C, which corresponds to 0.36 Tm^1^. Fine-grained AZ91 magnesium alloy exhibited superplastic behavior in the low temperature range of 150–250 °C (0.46–0.57 Tm)^19^. High carbon fine-grained (FG) steels (containing 1–2% C in weight %) were observed to exhibit superplasticity at 0.5–0.65 Tm and strain rate of ~10^−4^–10^−3^ s^20^,^21^. It is however, important that the forming steel must possess high thermal stability to retain the fine microstructure and provide outstanding mechanical properties in the final product^21^. Studies on superplasticity in steels at temperatures less than 0.5 Tm, especially in commonly used low carbon steels, have not been explored to the best of our understanding.

## Results

### Processing of low carbon NG/UFG microalloyed steel

**Figure 1:**
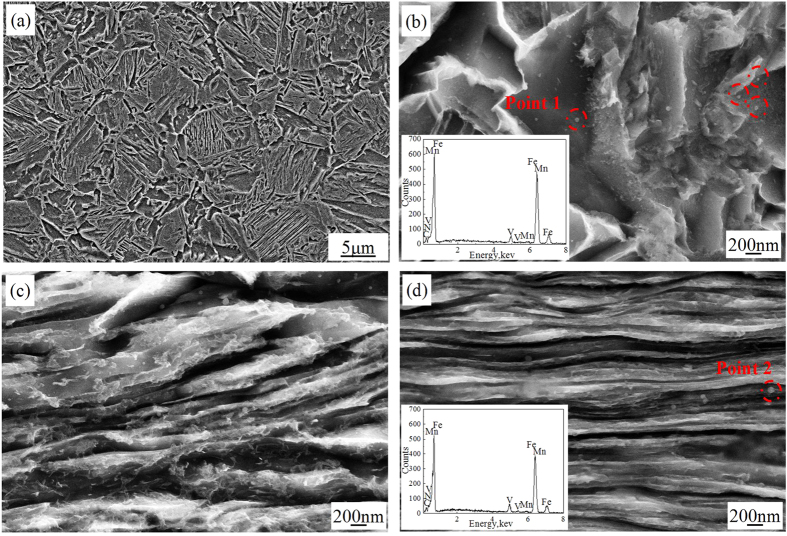
SEM micrographs of experimental steels subjected to water-quenching and cold-rolling. (**a**) reheating at 900 °C, followed by water quenching (**b**) water quenching steel with high magnification presenting V carbonitride at prior austenite grain boundaries (**c**) cold rolling of the water quenched steel with 1.6 mm thickness and (**d**) cold rolling of the water quenched steel with 0.9 mm thickness. The prior austenite grain size was 3–6 μm and the width of martensite plates was refined to 100–150 nm on cold-rolling to 0.9 mm thickness.

Scanning electron microscopy (SEM) micrographs of the experimental steels subjected to reheating at 900 °C followed by water quenching are presented in Fig. 1a. During reheating of the water-quenched martensitic microstructure, fine austenite nucleated at prior austenite grain boundaries and martensite lath. After the second quenching step, the microstructure consisted of fine martensite plates with the width of ~300–500 nm. The prior austenite grain size was 3–6 μm, and 20–30 nm VN precipitates were formed during holding at 900 °C, which were distributed within the prior austenite grains and martensite plates (Fig. 1b). The addition of N to V-microalloyed steels decreased the incubation period for the precipitation of V carbonitride and increased the volume fraction because of higher solubility product of V and N. According to the solubility product of V-N in austenite (equation 1)^22^, the complete dissolution temperature of VN in the experimental steel is 1102 °C. The pinning effect of VN decreased coarsening rate of prior austenite grains. After cold-rolling to 1.6 mm, the non-uniform martensite plates were obtained with width of 100–150 nm and 150–250 nm (Fig. 1c), while in the 0.9 mm thick cold-rolled plate, the martensite plates with width of 100–150 nm parallel to the rolling direction were obtained (Fig. 1d). Thus, superplasticity was studied in 0.9 mm thick cold-rolled plate because of fine and homogeneous original microstructure.


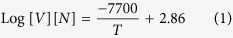


**Figure 2:**
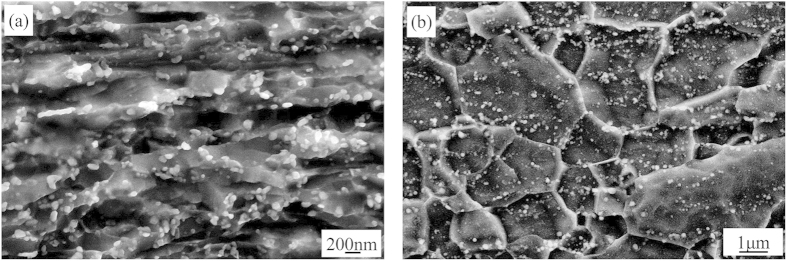
SEM micrographs of cold-rolled experimental steel subjected to annealing to obtain NG/UFG and FG microstructure. (**a**) 550 °C for 5 min and (**b**) 650 °C for 5 min. The NG/UFG steel was obtained on annealing at 550 °C and was characterized by a combination of NG/UFG ferrite and 50–80 nm fine precipitates, while FG steel with polygonal ferrite grains of 1–3 μm were obtained on annealing at 650 °C.

When cold-rolled plate of 0.9 mm thickness was annealed at 550 °C for 5 min, recrystallization occurred, and the microstructure consisted of UFG (100–300 nm) and few NG (<100 nm) ferrite grains, and 50–80 nm precipitates distributed along the grain boundaries. This microstructure is referred as NG/UFG steel (Fig. 2a). On annealing at 650 °C for 5 min, the majority of ferrite was coarsened to 1–3 μm, and few ferrite grains of submicron size were observed (Fig. 2b). This FG steel was used as a reference steel to study NG/UFG steel.

### The thermal stability of NG/UFG steel during isothermal holding

**Figure 3:**
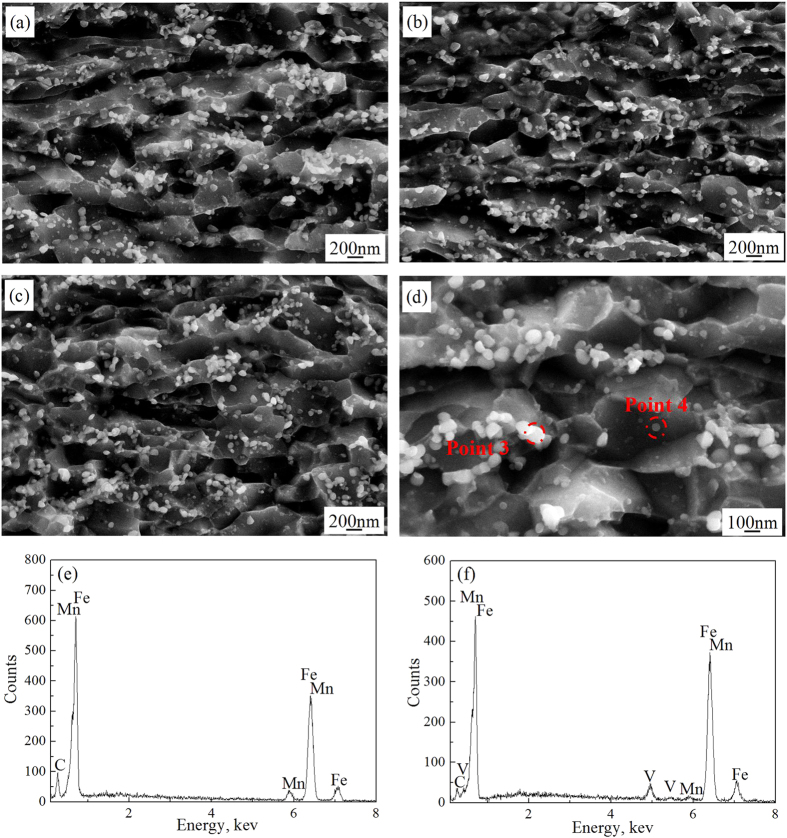
SEM micrographs of NG/UFG steel subjected to isothermal holding to determine thermal stability. Isothermally held at 500 °C for (**a**) 5 min, (**b**) 10 min, (**c**,**d**) 30 min, (**e**) chemical composition of precipitate at point 3, and (**f**) chemical composition of precipitate at point 4. The NG/UFG steel had higher thermal stability at 500 °C, and the morphology of elongated-shaped ferrite was altered to equiaxed ferrite on further recrystallization. Cementite of size 50–80 nm was distributed along the ferrite grain boundaries and 10–20 nm V(C,N) precipitates were present in the ferrite matrix.

**Figure 4:**
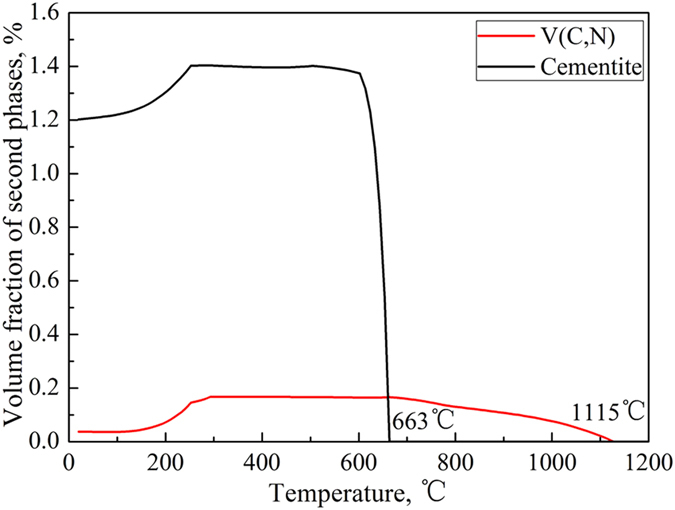
Volume fraction of second phases calculated by Thermocalc. The precipitation temperature of V(C,N) and cementite was 1115 °C and 663 °C, respectively.

The effect of isothermal holding time at 500 °C on thermal stability of NG/UFG steel was studied because this is the temperature at which tensile tests was carried out after annealing. For duration of 5 to 30 min, the NG/UFG ferrite was stable, and morphology of few grains was altered from elongated-shape to equiaxed demonstrating further recrystallization (Fig. 3a–c). Two types of precipitates were nucleated, 50–80 nm cementite distributed along ferrite grain boundaries and 10–20 nm V(C,N) precipitates (Fig. 3d–f). Based on the volume fraction of second phases calculated by Thermocalc, the precipitation temperature of cementite was 663 °C, and its volume fraction in equilibrium at 550 °C was 1.4%. Therefore, the cementite was completely dissolved during isothermal holding at 900 °C, and was precipitated during annealing. The precipitation temperature of V(C,N) was 1115 °C (consistent with 1102 °C calculated by solubility product of V-N in austenite), and its volume fraction in equilibrium at 550 °C was 0.17% (Fig. 4). It is proposed that the addition of a small amount of V was effective in enhancing the microstructural stability of UFG low carbon steel against thermal exposure because nanometer-sized V carbonitrides suppress grain growth, even if recrystallization were to occur^23^.

### Tensile properties of NG/UFG steel and comparison with FG steel

**Figure 5:**
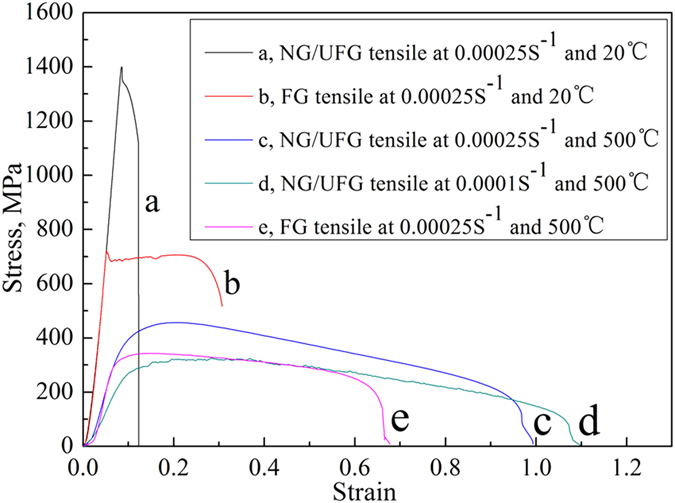
Stress-strain plots illustrating the deformation behavior of NG/UFG steel and FG steel. (**a**) NG/UFG straining at 0.00025 s^−1^ and 20 °C with tensile strength of 1400 MPa, elongation of 12%, and no work hardening, (**b**) FG straining at 0.00025 s^−1^ and 20 °C with yield strength of 681 MPa, tensile strength of 704 MPa, and elongation of 29%, (**c**) NG/UFG straining at 0.00025 s^−1^ and 500 °C with yield strength of 400 MPa, tensile strength of 457 MPa, and elongation of 95%, (**d**) NG/UFG straining at 0.0001 s^−1^ and 500 °C with yield strength of 278 MPa, tensile strength of 327 MPa, and elongation of 106%, (**e**) FG straining at 0.00025 s^−1^ and 500 °C with yield strength of 324 MPa, tensile strength of 343 MPa, and elongation of 57%.

NG/UFG steels were subjected to tensile tests at room temperature (20 °C) and moderately high temperature (500 °C) and compared with FG steel. Tensile straining NG/UFG steel at 0.00025 s^−1^ and 20 °C, the yield stress was 1400 MPa, and elongation was low at 12% because of absence of work hardening (black curve a in Fig. 5). While the FG steel indicated significant work hardening behavior, with yield strength, tensile strength, and elongation of 681 MPa, 704 MPa, and 29%, respectively (red curve b in Fig. 5). When the tensile test temperature was 500 °C, the NG/UFG steel tensile strained at 0.00025 s^−1^, the yield strength and tensile strength were reduced to 400 MPa and 457 MPa, respectively. An important characteristic was that the stress decreased slowly on reaching the peak stress, which depicts high elongation of 95% (blue curve c in Fig. 5). On decreasing the strain rate to 0.0001 s^−1^, the yield strength and tensile strength were further decreased to 278 MPa and 327 MPa, respectively, and the elongation was increased to 106%. During the softening process, non-uniform plastic stage was extended, where the NG/UFG steel continues to sustain load even at extraordinary strain of more than 1.0 (green curve d in Fig. 5). In contrast, FG steel strained at 0.00025 s^−1^ and 500 °C, was characterized by yield strength and tensile strength of 324 MPa and 343 MPa, respectively. The stress decreased rapidly implying abrupt necking, which resulted in reduced elongation of 57% (pink curve e in Fig. 5). The Young’s modulus decreased with increasing test temperature, consistent with the results obtained at high temperature in high strength structural steel, UFG steel, and austenitic stainless steel^24^,^25^,^26^. The elastic modulus was reduced with decreasing strain rate, which was also obtained in superplastic stress-strain plot of low carbon steel and FG INCONEL alloy 718SPF^27^,^28^.

## Discussion

The formation of NG/UFG grains is attributed to the original martensite structure and subsequent recrystallization process. Tsuji^29^,^30^,^31^ proposed that lath martensite has a three-level hierarchy in this morphology: (I) Lath - single crystal of martensite including high density of lattice defects, (II) Block - aggregation of laths with the same crystallographic relationship, and (III) Packet - aggregation of the blocks having identical habit plane. An austenite grain is sub-divided into several packets and blocks during martensite transformation. Majority of the block boundaries and packet boundaries are high-angle boundaries. Thus, martensite has a fine-grained structure in the as-transformed state. Takaki^32^ proposed that cold rolling of martensitic steel introduces slip bands into the matrix and this results in the destruction of lath martensitic structure and the formation of lamellar dislocation cell structure around slip bands. The volume fraction of such a damaged martensite increases with increasing deformation. The undamaged lath martensitic structure was rarely observed and dislocation density was one order of magnitude higher in specimens subjected to severe cold reduction greater than 80%, leading to increase in nucleation rate of recrystallized ferrite grains. It was also proposed^29^,^32^,^33^ that the blocks and packets of martensite have angular and rugged shape. Such high density of high-angle boundaries and complicated shape of blocks and packets would produce strong constraint effect during plastic deformation. The constraint causes inhomogeneous deformation (grain sub-division) resulting in ultrafine deformed microstructure with large local misorientation. During the annealing process, the deformation-induced damaged martensitic structure transformed into NG/UFG ferritic grains via recovery process involving escape of interstitial C atoms and annihilation of dislocations (also called *in situ* recrystallization or continuous recrystallization). At the same time, the cementite formed due to supersaturated solid solution of C atoms in the damaged martensitic structure. The nano-scale cementite and V(C,N) precipitates increased the thermal stability of NG/UFG steel because their pinning effect inhibited grain growth.

NG/UFG steel exhibited unusual deformation behavior compared with the FG steel, and tensile ductility was decreased from 29% to 12% at 0.00025 s^−1^ and 20 °C. Song^7^ proposed that this decrease in tensile ductility at room temperature for majority of UFG steels, especially single phase steels, was mainly due to dynamic recovery and plastic instability. First, dynamic recovery as a softening mechanism is able to reduce the apparent work hardening rate. During deformation, dislocations are trapped at grain boundaries. The kinetics of dynamic recovery are associated with the spreading of trapped lattice dislocations to grain boundaries especially in UFG steels^34^,^35^,^36^. For UFG steel, the time for dislocations to move to grain boundaries is shorter than the time of tensile test^37^. This decrease in dislocation density reduces accumulation of dislocations inside the grains, and consequently leads to less work hardening when compared with corresponding steels of coarse grain size. Second, the decrease in tensile ductility can be explained in terms of plastic instability, which initiates necking due to localized deformation^38^,^39^. Ultra grain-refinement greatly increases the flow stress of steels, especially during the early stages of plastic deformation. Grain refinement also leads to reduced work hardening capacity. As a result, plastic instability (necking) occurs at an early stage during tensile test, which results in limited uniform elongation in UFG steels. Moreover, in NG/UFG steels, necking occurs during deformation of Lüders band after the yield drop, and work-hardening disappears^40^.

The elongation of NG/UFG steel at 0.00025 s^−1^ and 500 °C was greatly enhanced from 57% to 95% in comparison to FG steel. Zhilyaev^41^ and Nieh^42^ proposed that grain boundaries play an active role during high-temperature deformation, and the increase of effective diffusion coefficient in NG/UFG materials may approach values several orders of magnitude higher in comparison to their coarse-crystalline counterpart. In NG/UFG steels, the deformation mechanism of grain boundary sliding begins to operate, and it is necessary for an accommodation process to accompany grain boundary sliding and govern the kinetics of superplasticity. This accommodation process might be grain-boundary migration, recrystallization, diffusional flow, or dislocation slip. The elongation was further increased to 106% at lower strain rate of 0.0001 s^−1^ and 500 °C, representative of low temperature superplastic behavior. It is proposed^43^ that as the strain rate is decreased, the average cavity size and roundness coefficient increases. The average roundness coefficient is indicative of the degree of diffusion associated with cavity growth. A roundness coefficient of one indicates a perfect circular shape, while a smaller value of roundness coefficient relates to cavity elongation. The increase in roundness coefficient with decreasing strain rate suggests that cavity growth by diffusion is becoming increasingly significant.

The stress-strain plot at 0.0001 s^−1^ and 500 °C appeared serrated flow, which was also observed in superplastic stress-strain plot of ultrahigh-carbon steel^21^,^44^, aluminum alloy^1^ and magnesium alloy^19^, which is presumably related to dynamic strain aging (DSA) or discontinuous recovery and periodic recrystallization involving stress relaxation and transfer during deformation. DSA is generally attributed to additional resistance to dislocation motion produced by the mobility of solute atoms that can diffuse to dislocations above a certain temperature^27^,^45^. The serrations resulted from the recurrent pinning of dislocations, such that the dislocations are stopped by obstacles such as precipitates and diffusing solute atoms^46^. A key feature of thermally activated dislocation motion is that the dislocation moves in a jerky manner because the dislocations spend majority of their time to interact with the local obstacles, such as forests of dislocations, vacancies, and solute atoms^47^. Moreover, DSA effect increased with decrease in strain rate^48^.

In summary, we have observed that low-carbon steel with NG/UFG ferrite and 50–80 nm cementite has high thermal stability at 500 °C with elongation exceeding 100% (<0.5 T_m_). An important implication of the study is that the low strength during hot processing is favorable for decreasing the spring back and minimize die loss. The low temperature superplasticity is satisfactory to forming complex components with low energy consumption and weaker surface oxidation.

## Materials and Methods

The experimental steel was melted in vacuum induction furnace and cast as 50 kg ingot. The nominal chemical composition of the experimental steel in wt% was 0.1 C, 0.16 Si, 1.55 Mn, 0.02 Al, 0.1 V, 0.018N, 0.5–1 Ni, and balance Fe. The 45 mm thick slab was heated to 1200 °C for 3 h. After air-cooling to 920 °C, the slab was rolled via 7 passes to a plate of 5.5 mm on a rolling mill with roll diameter of 450 mm. The finish rolling was controlled at 805 °C, and then the experimental steels were accelerated cooled to room temperature. The microstructure consisted of polygonal ferrite, acicular ferrite, and granular bainite. The martensite phase was absent because deformation inhibited transformation of martensite.

The low carbon NG/UFG V-N microalloyed steel was processed through a combination of cold-rolling and annealing of martensite. In the first step, the 5.5 mm thick and 50 mm width steel was reheated at 900 °C for 5 min in a chamber electric furnace, and it took 6 min to reach the isothermal temperature. Then, the plate was water quenched to room temperature at a cooling rate of 90 °C/s to obtain martensitic microstructure. Next, in the second step, after removing the scale, the steel of 5 mm thickness was again cold rolled to 1.6 mm and 0.9 mm in thickness, respectively, with 68% and 82% total reduction, and 0.3–0.5 mm reduction was carried out in each pass depending on the rolling resistance. The tensile samples were annealed at 550 °C and 650 °C, respectively, in a tubular furnace, with heating rate of 10 °C/s. Subsequently the specimens was water-quenched to room temperature to avoid the microstructural coarsen.

### Characterization of microstructure

The specimens for microstructural studies were polished to mirror finish using standard metallographic procedure involving use of SiC paper of different surface roughness, followed by final polishing using diamond paste of particle size of 2.5 μm. The polished specimens were etched with a 4 volume percent nital solution and observed using a Zeiss Ultra 55 SEM with an accelerated voltage of 15 kV. The 1/4 thickness was observed to represent the bulk. Moreover, the high microstructural homogeneity along thickness was obtained due to large total cold rolling reduction. The chemical composition of the precipitates was determined by energy-dispersive X-ray spectroscopy (EDX) with an accelerating voltage of 15 kv and instant probe size of smaller than 10 nm. The theoretical calculations concerning evolution of various phases with temperature, such as V(C,N) and cementite, were studied using Thermocalc combined with TCFE6 database for thermodynamic calculation in equilibrium.

### Tensile tests

Tensile tests were carried out using dog-bone shaped samples of 10 mm gage length and 5 mm gage width at room temperature and 500 °C at strain rate of 0.0001 s^−1^ and 0.00025 s^−1^, respectively, using Shimadzu AG-X universal testing machine. An extensometer was used for the room temperature tests. For tensile at 500 °C, the specimens were heated to 500 °C for 5 min prior to loading. The tensile data presented is an average of five measurements for each test condition.

## Additional Information

**How to cite this article**: Hu, J. *et al.* Low temperature superplasticity and thermal stability of a nanostructured low-carbon microalloyed steel. *Sci. Rep.* **5**, 18656; doi: 10.1038/srep18656 (2015).
